# A 17-year time-series of fungal environmental DNA from a coastal marine ecosystem reveals long-term seasonal-scale and inter-annual diversity patterns

**DOI:** 10.1098/rspb.2022.2129

**Published:** 2023-02-08

**Authors:** Nathan Chrismas, Ro Allen, Michael J. Allen, Kimberley Bird, Michael Cunliffe

**Affiliations:** ^1^ Marine Biological Association, The Laboratory, Citadel Hill, Plymouth PL1 2PB, UK; ^2^ College of Life and Environmental Sciences, University of Exeter, Exeter EX4 4PY, UK; ^3^ Plymouth Marine Laboratory, Prospect Place, Plymouth PL1 3DH, UK; ^4^ School of Biological and Marine Sciences, University of Plymouth, Plymouth PL4 8AA, UK

**Keywords:** marine fungi, plankton, seasonality, marine ecosystem

## Abstract

Changing patterns in diversity are a feature of many habitats, with seasonality a major driver of ecosystem structure and function. In coastal marine plankton-based ecosystems, seasonality has been established through long-term time-series of bacterioplankton and protists. Alongside these groups, fungi also inhabit coastal marine ecosystems. If and how marine fungi show long-term intra- and inter-annual diversity patterns is unknown, preventing a comprehensive understanding of marine fungal ecology. Here, we use a 17-year environmental DNA time-series from the English Channel to determine long-term marine fungal diversity patterns. We show that fungal community structure progresses at seasonal and monthly scales and is only weakly related to environmental parameters. Communities restructured every 52-weeks suggesting long-term stability in diversity patterns. Some major marine fungal genera have clear inter-annual recurrence patterns, re-appearing in the annual cycle at the same period. Low relative abundance taxa that are likely non-marine show seasonal input to the coastal marine ecosystem suggesting land–sea exchange regularly takes place. Our results demonstrate long-term intra- and inter-annual marine fungal diversity patterns. We anticipate this study could form the basis for better understanding the ecology of marine fungi and how they fit in the structure and function of the wider coastal marine ecosystem.

## 1. Introduction

Seasonality is a characteristic feature of many terrestrial and aquatic ecosystems, with diversity and abundance of taxa changing over an annual cycle in a generally recurring year-on-year pattern that is controlled, in part, by prevailing environmental parameters such as temperature and light availability. Seasonal-scale changes in diversity and abundance regulate major ecosystem processes and are impacted by ongoing climate change and other anthropogenic pressures.

Seasonality in marine plankton communities has been known for some time [1] and has a regulatory role in marine ecosystem processes, such as carbon cycling and food web provision. Historically, temporal assessment of marine plankton has focused on morphologically distinct groups (e.g. zooplankton and some phytoplankton such as diatoms) because these taxa are readily identified with microscopes. The relatively recent application of environmental DNA (eDNA)-based approaches (i.e. seawater sampling by filtration, DNA extraction and subsequent sequencing) has improved the assessment of plankton diversity by allowing the characterization of entire communities, including groups previously overlooked by microscope-based observation because taxa are too small (e.g. picoeukaryotes), morphologically indistinct (e.g. bacterioplankton) or somehow cryptic (e.g. intracellular parasites).

Long-term time-series studies are critical to understanding plankton temporal dynamics and ecology [2]. Regular plankton sampling at fixed spatial points (i.e. sampling stations or observatories) alongside quantification of concurrent environmental parameters provide a platform for understanding seasonality. A few long-term (greater than 10 years) and temporally well-resolved eDNA-based studies have been conducted to assess marine plankton dynamics through time, which have so far been focused on bacterioplankton and protists [3–5]. Long-term eDNA-based time-series studies conducted in temperate marine ecosystems have shown intra-annual patterns in microbial plankton diversity at the community level [3,4] and within individual taxa [5]. Seasonal-scale changes in plankton communities are associated with physico-chemical environmental parameters (e.g. temperature, nutrient availability) as well as other less well-understood processes that likely include biotic interactions (e.g. predator–prey, parasite–host) and dispersal or drift between and within ecosystems.

Fungi are widespread and active members of marine plankton communities and are prevalent from coastal waters to the open ocean [6]. As with other microbial plankton groups, the assessment of marine fungal diversity relies on eDNA-based approaches. Current understanding of marine fungal diversity in an environmental context is so far based principally on spatial eDNA studies. For example, an assessment of global marine fungal diversity has shown that most communities are dominated by the phyla Ascomycota and Basidiomycota, with evidence of biogeographic patterns in diversity at the basin scale [7].

While a small number of short-term temporal studies of marine fungal diversity have been conducted [8–10], fungi have not been considered in terms of long-term temporal dynamics compared to other plankton groups. As a result, we lack an understanding of the persistent intra-annual (including seasonal-scale) and inter-annual diversity patterns of marine fungi at both community and individual taxon levels. In this study, we used a 17-year eDNA time-series with weekly sampling resolution from the long-term ecosystem research site Station L4 in the western English Channel off Plymouth (UK). We focused on Ascomycota and Basidiomycota because they are the two major marine fungal groups generally in the oceans [7] and specifically at Station L4 [8]. In this study, we addressed the following questions: What are the long-term intra- and inter-annual patterns in marine fungal diversity? Which environmental parameters are associated with long-term changes in marine fungal diversity? Do marine fungi follow similar long-term temporal diversity patterns as other microbial plankton groups? To what extent do non-marine fungi contribute to eDNA-based assessments of coastal marine fungal diversity? Our approach was to explore fungal diversity at both the community (i.e. community structure and alpha diversity) and individual taxon (i.e. genus) level, as shown to be important in the assessment of marine protist seasonality [5]. The goal of our work is to provide greater insight into marine fungi and to support the development of a more complete view of seasonality in coastal marine plankton-based ecosystems.

## 2. Methods

### (a) Seawater environmental DNA sampling and environmental parameters

**Figure 1.**
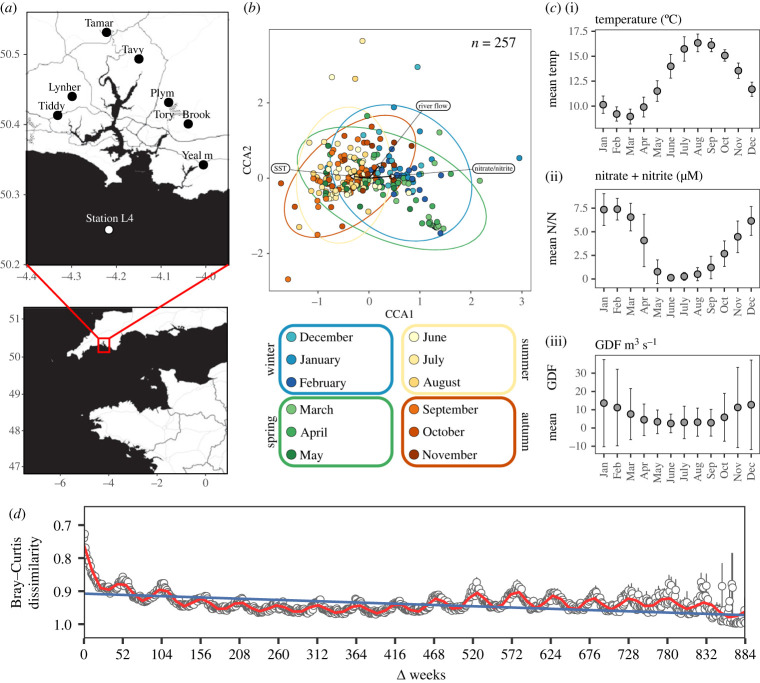
(*a*) Geographical location of the long-term ecosystem research site Station L4 and hydrological stations on the Tamar estuary catchment. (*b*) CCA of fungal communities at Station L4 based on 257 samples (greater than 1000 reads per sample) collected between January 2002 and December 2018. Only significant driving environmental variables are shown. Ellipses indicate 95% confidence intervals around the centroid of each grouped season. (*c*) Monthly patterns of (i) temperature, (ii) nitrate + nitrite and (iii) mean GDF from all Tamar catchment hydrological stations. (*d*) Bray–Curtis dissimilarities for each pair of samples in the dataset 1–884 weeks apart. Bray–Curtis dissimilarities cycle periodically, being lowest at 1-year intervals indicating annually repeating community composition.

Sampling was carried out at the long-term ecosystem research site Station L4 in the western English Channel off Plymouth (UK) (50° 15.00′ N, 4° 13.02′ W) (figure 1*a*) between January 2002 and December 2018 on near weekly cruises (electronic supplementary material, File S1) onboard the RV *Plymouth Quest*. Seawater samples were collected from surface water using a Niskin bottle attached to a CTD rosette. One litre seawater sub-samples were filtered onto 0.45 µm cellulose-nitrate filters and stored at −80°C.

Associated environmental parameters, including sea surface temperature (SST), salinity and nutrient concentrations were collected simultaneously to seawater eDNA samples as part of the Western Channel Observatory (WCO) (https://www.westernchannelobservatory.org.uk). River flow (Gauged Daily Flow (GDF)) and rainfall were used as proxies for estuarine impact and were obtained from hydrological stations on the Tamar estuary catchment adjacent to Station L4 from the National River Flow Archive (https://nrfa.ceh.ac.uk/) (figure 1*a*). Where GDF and rainfall sampling dates did not exactly match those of seawater, the closest matching dates were found and assigned using custom R scripts (electronic supplementary material). Samples without matching dates within less than 3 days were discarded. All metadata used in this study are also available in the electronic supplementary material, File S1.

### (b) Environmental DNA extraction and metabarcoding

eDNA was extracted from the cellulose-nitrate filters in a pre-cleaned laminar flow hood using the ZymoBIOMICS DNA Miniprep Kit (Zymo Research). Filters were cut up using a sterile scalpel and transferred to a ZymoBIOMICS lysis tube containing 700 µl kit lysis solution. Samples were bead beaten three times for one minute with 5 min intervals on ice using the FastPrep-24 (MP Bio) bead beater at 10 ms. Manufacturer's protocols were then followed with a final elution volume of 100 µl. With each batch of extractions (13 batches), a blank sample (i.e. no filter) was also processed to determine possible extraction process or kit-associated contaminants. The ‘Fungi-specific’ internal transcribed spacer (ITS) region was amplified using the PCR primers ITS1F-ITS2 [11] according to Earth Microbiome Project (EMP) protocols [12] and sequenced on the Illumina Miseq platform.

### (c) Sequence processing

A total of 48316978 raw ITS1 sequences were returned and primers trimmed using CutAdapt v1.5 [13]. Trimmed sequences were processed using the DADA2 pipeline [14] according to protocols described in Pauvert *et al*. [15] keeping only forward reads so that reads that failed to merge due to variation in sequence length were not discarded. Resulting amplicon sequence variants (ASVs) were first classified using the UNITE database [16]. Putative ‘Fungi’ ASVs were then manually curated by performing BLAST searches against the NCBI nr database (10 November 2020) (e-value = 0.001). Local BLAST searches were used to generate the 10 best hits (electronic supplementary material, file S2) and manually inspected to establish a consensus identity. In cases where a single genus dominated hits for an individual sequence, this was then assigned as the genus for that sequence. If there was no consensus genus, the same processes were applied to higher taxonomic ranks. If no consensus could be determined, a more extensive search comparing greater than 20 hits was carried out before indeterminate sequences were classified as unknown. Specific identity and query coverage thresholds were not used due to high levels of variation within the length of some ITS sequences [17]. All sequence assignments can be found in the electronic supplementary material, file S3. Based upon this manual classification, metazoan, protist and unknown sequences were excluded from further analysis. For the confirmed fungal genera, each genus was searched against the FUNGuild database [18] and assigned growth form (e.g. yeast), trophic mode (e.g. saprotroph) and guild (e.g. pathogen). Further downstream analysis was conducted using phyloseq [19]. Due to high numbers of non-fungal ITS sequences, total fungal reads for many samples were low (i.e. < 10 000 reads). To maximize the number of samples retained, a low retention threshold was used for alpha and beta diversity analyses, discarding samples that contained less than 1000 reads and rarefying to 1008 reads per sample in a trade-off between capturing complete diversity and maintaining high temporal resolution. Unrarefied reads were used for all single taxon analyses. Sequences are deposited in NCBI Sequence Read Archive (SRA) under BioProject PRJNA865278.

### (d) Statistical analysis

Analyses were performed on the complete fungal dataset using the vegan package [20] in R v.3.6.3 [21]. Shannon and observed diversity (i.e. number of ASVs) were calculated using the *estimate_richness* function, and Pielou's evenness was calculated as Shannon/observed_log_. Additionally, Chao1 was included as a richness estimator as it is more robust when sequencing depth is limited. Comparison of alpha diversity metrics between seasons (defined as Northern Hemisphere meteorological seasons (spring = March, April and May; summer = June, July and August; autumn = September, October and November; winter = December, January and February), months and weeks was performed using a Kruskal–Wallis with pairwise Wilcoxon *post hoc* test. Permutational multivariate analysis of variance (PERMANOVA) of communities at seasonal, monthly and weekly scales was carried out on a Bray–Curtis distance matrix using the *adonis* function. Further pairwise comparisons were performed using *pairwise.adonis* [22].

To investigate the relationship of fungal community composition to environmental parameters (electronic supplementary material, file S1), we performed a canonical correspondence analysis (CCA) on the Bray–Curtis distance matrix. Patterns in inter-annual community dynamics over the time-series were calculated according to methods as described in Furhman *et al*. [23]. Bray–Curtis dissimilarities were calculated for each pair of samples in the dataset *n* (1–884) weeks apart e.g. data points one, 26 and 52 represent Bray–Curtis dissimilarity of communities one week, six months and 1 year apart, respectively.

To determine recurrence patterns of key individual taxa, we applied the recurrence index (RI) approach developed by Giner *et al*. [5] on all taxa constituting greater than 1% of fungal reads. First, autocorrelation function (ACF) analysis was performed for each of the 16 taxa greater than 1%. ACF values for each taxon were summed across the entire dataset (RF) and subsequently randomized 1000 times. Summed randomized values (RF_random_) were then compared to RF values to calculate RI with RI = RF/RF_random_. Taxa were classed as recurrent if (i) RF was significantly higher than RF_random_ and (ii) RI was greater than 1.09. To establish whether certain individual taxa are associated with seasons, we used INDicator VALue (IndVal) analysis with the *indicspecies* package [24] (perm = 999). Trophic mode and growth form for each of the recurrent taxa were predicted by searching against the FunGuild database [18].

## 3. Results

### (a) Fungal environmental DNA overview

Taxonomy was assigned to 19 127 649 reads, of which 48.4% (9 252 590 reads) were classified as ‘Fungi’ using the UNITE database and were clustered into 1979 ASVs. Following manual curation of the UNITE-determined ASVs, 65.5% (1298 ASVs) were confirmed fungal with 10.1% (201 ASVs) non-fungal (other), including hydrozoans, chaetognaths and protists, and 24.2% (480 ASVs) indeterminate, i.e. no clear taxonomic affiliation (unknown) (electronic supplementary material, figure S1*a*). Of the manually confirmed 1298 fungal ASVs, 51% (662 ASVs) were assigned to Ascomycota, 28.7% (372 ASVs) were Basidiomycota, 1.2% (15 ASVs) were Mucoromycota, 0.5% (7 ASVs) were Glomeromycota and 0.07% (1 ASV) were Neocallimastigomycota. The remaining 18.5% (241 ASVs) could not be confidently classified below kingdom level (electronic supplementary material, figure S1*b*). The manually confirmed 1298 fungal ASVs were the focus of this study.

### (b) Community structure

Consolidation of the 17-year time-series showed that fungal community structure in surface coastal marine waters varies throughout the course of a year and at different temporal scales (figure 1*b*). At the seasonal scale (winter, spring, etc.), communities were different to each other (PERMANOVA, *F* = 5.63, *p* = 0.001) (electronic supplementary material, table S1). In terms of the monthly scale progression through an annual cycle, November, December and January had similar community structures to each other but differed to February, March and April which were also similar. The year then progressed with communities being similar in adjoining months (i.e. May–June, July–August, September–October and November–December) but different from all other months (PERMANOVA, *F* = 2.9, *p* = 0.001) (electronic supplementary material, table S2). At the weekly scale, the pattern of community similarity/dissimilarity over the time-series became complex and began to break down (PERMANOVA, *F* = 1.32, *p* = 0.001) (electronic supplementary material, table S3).

We explored the relationship of intra-annual changes in community structure to the available concurrent environmental parameters at Station L4 and river flow to the region. Changes in environmental parameters averaged over the time-series were typical for a temperate coastal marine shelf sea, which included an increase in SST from a minimum in late winter/early spring to a maximum in late summer/early autumn, and macronutrient (nitrate/nitrite, silicate and phosphate) depletion after winter likely driven by the spring phytoplankton bloom. River flow was variable and, as expected, minimal during the summer (figure 1*c*). Surface seawater temperature, nitrate/nitrite availability and river flow showed a relationship with the winter/spring (riverflow; *F* = 1.52, *p* ≤ 0.05 and nitrate/nitrite *F* = 1.43, *p* ≤ 0.001) and summer (SST; *F* = 1.39, *p* ≤ 0.05) communities. Even though river flow, nitrate/nitrite availability and SST are the best variables tested to describe fungal community structure over the time-series, these environmental parameters only explain approximately 3% of the total variation in the dataset.

In line with long-term studies of bacterioplankton and protist community structure based on metabarcoding analysis of eDNA samples [3,5], Bray–Curtis dissimilarity between individual samples was used to assess inter-annual patterns of fungal community structure. Bray–Curtis dissimilarity was high between samples, however, despite this, seasonality was observed, and comparison between the samples showed a sinusoidal recurring 52-week scale pattern (figure 1*d*), further suggesting that congruent communities over annual cycles have similar structures at reciprocal times of the year (as discussed above). The recurring 52-week scale pattern over the 17 years showed a small decay in Bray–Curtis dissimilarity.

### (c) Alpha diversity

**Figure 2.**
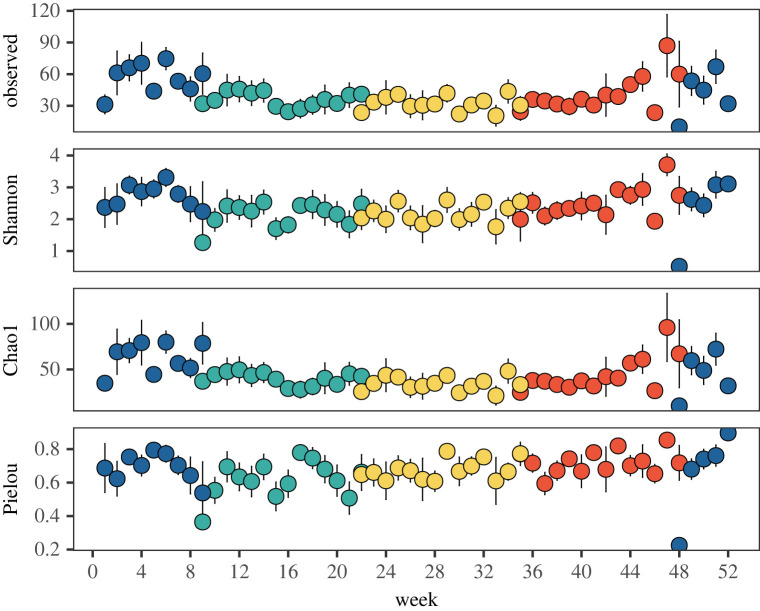
Annual diversity indices of fungal communities at Station L4. Points are coloured by season (blue = winter, green = spring, yellow = summer, red = autumn). Diversity is increased in winter under all measures of diversity (observed, Shannon, Chao1), whereas no difference was seen in Pielou's evenness.

Alpha diversity also showed intra-annual seasonal-scale changes (figure 2) (observed *χ*^2^ = 17.26, d.f. = 3, *p* < 0.001; Shannon *χ*^2^ = 15.006, d.f. = 3, *p* = 0.002; Chao1 *χ*^2^ = 25.73, d.f. = 3, *p* < 0.001). All estimates showed greatest diversity in the winter, followed by a reduction in the spring (pairwise Wilcoxon test *p* < 0.05). No significant difference in Pielou's evenness was detected (figure 2), indicating that the elevated alpha diversity in winter is the result of increased species richness.

### (d) Genus-level assessment

A total of 351 genera were manually identified from the eDNA-based survey of the coastal marine site (electronic supplementary material, figure S2). Focusing on genera with greater than 1% fungal reads, we investigated inter-annual recurrence across the 17-year time-series using the RI previously developed for marine protists [5]. Most of the genera greater than 1% fungal reads have been included in at least one previous ‘list of marine fungi’ (electronic supplementary material, table S4) and are assigned as either yeast or microfungus growth forms. Of the 16 genera assessed, 10 (62.5%) displayed evidence of inter-annual recurrence with a RI greater than 1.09 and a RF significantly higher than RF_random_ (electronic supplementary material, table S4).

**Figure 3.**
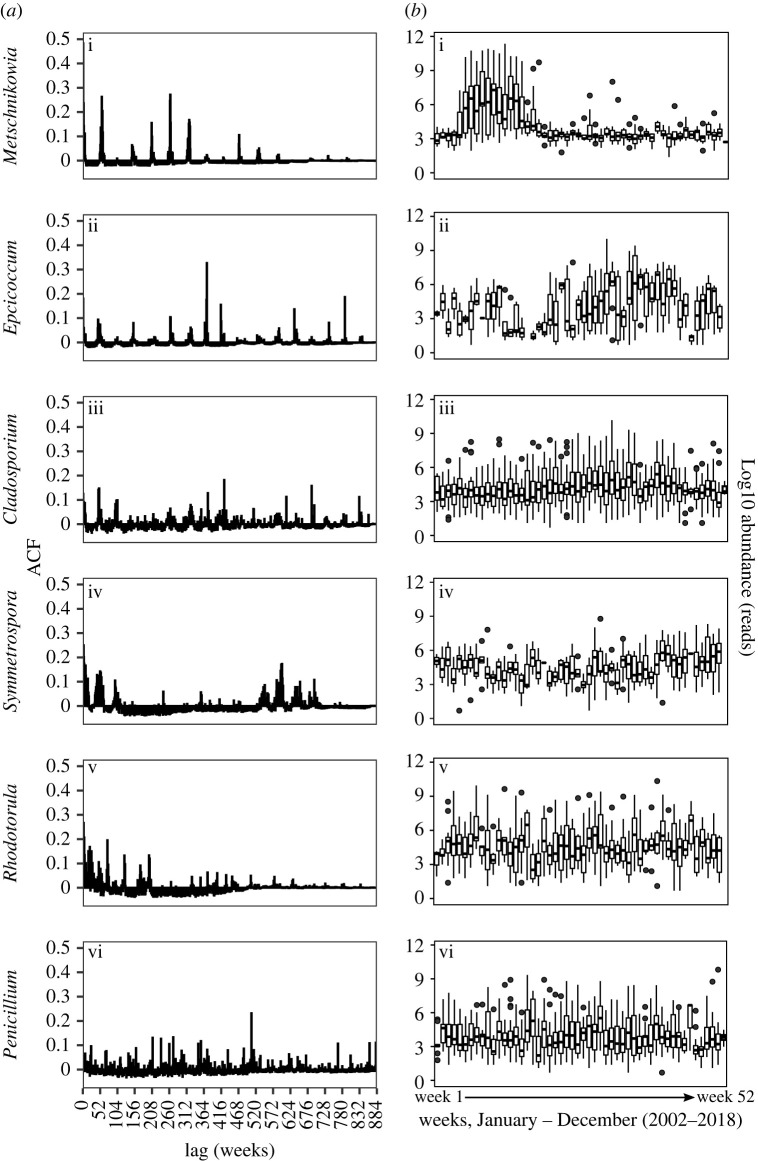
(*a*) (i–vi) ACF plots of abundance of six select fungal genera at Station L4 showing annual (i,ii), persistent (iii), occasional (iv,v) and random (vi) recurrence patterns. (*b*) (i–vi) annual abundance patterns of six select fungal genera at Station L4.

Patterns of inter-annual recurrence varied between the genera (figure 3; electronic supplementary material, figure S3). For example, *Metschnikowia* (yeast) and *Epicoccum* (microfungus) showed distinctive recurrence patterns once each year across the entire time-series (figure 3*a*(i) and (ii)), with increases in relative abundance around March and September, respectively (figure 3*b*(i) and (ii)). Other genera, such as *Cladosporium* (microfungus) and *Symmetrospora* (yeast) initially showed distinctive recurrence patterns once each year for a few years (less than 3 years) before the patterns became irregular (figure 3*a*(iii) and (iv)), with both genera having less obvious increases in relative abundance over an averaged year (figure 3*b*(iii) and (iv))*.* For some genera, such as *Rhodotorula* (yeast) and *Penicillium* (microfungus), there was no clear recurrence pattern through the time-series (figure 3*a*(v) and (vi)), with their relative abundance in the community appearing stable over an averaged year (figure 3*b*(v) and (vi)). Some genera only reoccurred for part of the time-series, such as *Sarocladium* (microfungus) and *Simplicillium* (microfungus) (electronic supplementary material, figure S3).

**Figure 4.**
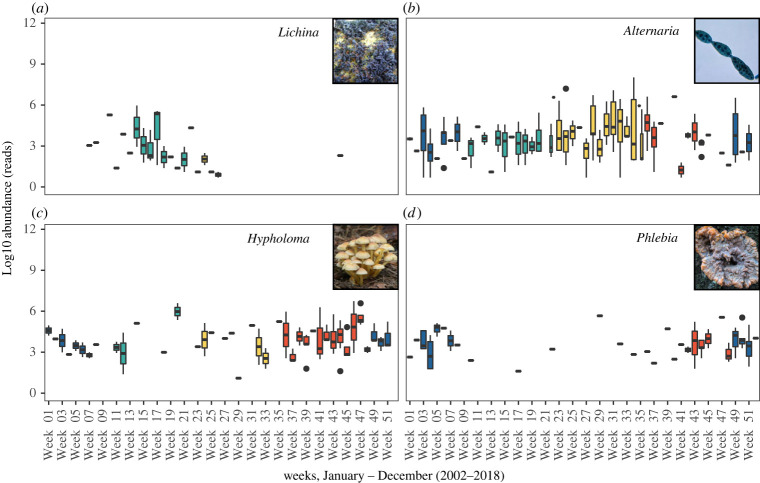
Abundance patterns of four select fungal genera dominant during (*a*) spring (*Lichina*), (*b*) summer (*Alternaria*), (*c*) autumn (*Hypholoma*) and (*d*) winter (*Phlebia*) as determined by IndVal analysis, representing both marine and terrestrial lineages. Bars are coloured by season (blue = winter, green = spring, yellow = summer, red = autumn). All photographs Wikimedia Commons.

All identified genera (i.e. across the relative abundance range in the dataset) were assessed to determine significant seasonal-scale differences over the time-series, which showed that 42 genera where associated with either winter (14 genera), spring (13 genera), summer (7 genera) or autumn (8 genera) (electronic supplementary material, table S5). Many of the genera displaying significant seasonality are terrestrial macrofungi typically associated with forest ecosystems, and that have corticioid, agaricoid, pezizoid or polyporoid growth forms (determined via FunGuild). The macrofungi genera were generally associated with winter, spring and autumn communities (electronic supplementary material, table S5; figure 4) when river flow is greatest because of increased rainfall (figure 1*c*(iii)). It is important to note that these likely terrestrially derived macrofungi genera were represented at low relative abundances in the dataset (electronic supplementary material, figure S2). Conversely, the marine-associated genera already discussed above, *Metschnikowia* and *Cladosporium*, were well represented in the dataset, and were significantly associated with spring and summer respectively, matching their averaged intra-annual distribution patterns (figure 3). Finally, one other taxon of note is the marine lichen genus *Lichina*, which has two UK species (*L. pygmaea* and *L. confinis*). *Lichina* grow on wave-exposed rocky shores and are abundant along the coastline close to Station L4 all year round [24,25], yet they appear in the time-series dataset only in the spring and mainly between March and May (electronic supplementary material, table S5, figure 4).

## 4. Discussion

The aim of this study was to use a 17-year coastal marine surface water eDNA time-series to determine long-term intra- and inter-annual patterns in fungal diversity. We show that there are intra-annual changes in community structure at seasonal and monthly scales with similarity in community structure peaking at 52-week intervals, supporting the conclusion that coastal marine fungal communities cycle through, and restructure to, similar patterns year-on-year over decadal scales.

Comparable long-term and temporally well-resolved eDNA-based studies of other plankton groups have shown analogous patterns in community structure. Surface water bacterioplankton at the San Pedro Ocean Time-series (SPOT) off the coast of Southern California (USA) showed similar sinusoidal patterns with maximum Bray–Curtis similarities at 52 weeks [3]. Surveys at the SOMLIT-Astan station in the western English Channel off Roscoff (France) and the Blanes Bay Microbial Observatory (BBMO) near Barcelona (Spain) in the northwestern Mediterranean Sea assessed temporal dynamics in protist diversity, both showing long-term seasonality in community structure as shown here for fungi [4,5]. Across the time-series, we observed a small inter-annual decay in Bray–Curtis dissimilarity, also shown with surface water bacterioplankton at the SPOT [3] and protists at the SOMLIT-Astan station [4], but not with protists at BBMO [5]. Inter-annual decay in community structure similarity suggests some degree of turnover taking place caused by immigration of taxa or other ecological selection process.

As part of the recurrent changes, we show that alpha diversity had a consistent intra-annual pattern over the 17-year time-series, being highest in the winter and relatively reduced in the spring/summer. These findings are similar to a 3-year study at the Pivers Island Coastal Observatory site where fungal ITS metabarcoding showed winter peaks in diversity and reduction in the summer [10]. Their study also used fungal 18S rRNA gene qPCR as a proxy for abundance which was minimal in winter when diversity is highest and peaked in the summer when diversity was lowest, suggesting an inverse relationship between fungal alpha diversity and abundance in coastal marine waters [10]. Intra-annual cycles of alpha diversity maxima and minima coinciding with when abundance is lowest and highest, respectively, are also observed with bacterioplankton [3,26] and protists including phytoplankton [4,5].

As well as showing changes in community-level diversity (community structure and alpha diversity), we also observed long-term patterns within individual taxa at the genus level. We identified genera that recurred in the time-series with different patterns of recurrence ranging from annually (e.g. *Metschnikowia*) to no obvious pattern (e.g. *Cadophora*), and consistently across the entire 17-year time-series (e.g. *Epicoccum*) to only part of the time-series (e.g. *Sarocladium*). We are not aware of any comparable analysis of marine fungi.

Understanding these taxon-specific patterns contribute to establishing the roles of marine fungi in an ecological context. For example, the ascomycete yeast *Metschnikowia* which increases annually in spring (figure 3) has been found infecting marine copepods [27]. Marine *Metschnikowia* isolates under laboratory conditions are also capable of infecting and killing the model zooplankton *Daphnia magna* [28], and *Metschnikowia–*zooplankton parasite–host interactions occur in freshwater ecosystems [29]. The seasonal zooplankton cycle at Station L4 is characterized by an annual increase in abundance during the spring that is dominated by copepods [30]. It is possible that the observed spring increase in *Metschnikowia* results from *Metschnikowia–*zooplankton parasite–host interaction, potentially with copepods, the prevalence and impact of which on ecosystem structure and function is yet to be determined.

A second example is the microfungus *Cladosporium*, one of the major genera throughout the time-series which shows regular recurrence each year and increases in relative abundance during the late summer/early autumn, corresponding to the second major phase of phytoplankton production [31]. Planktonic marine *Cladosporium* occupy niches associated with phytoplankton by using phytoplankton-produced high molecular weight organic matter via degradation with extracellular hydrolases [32]. DNA Stable Isotope Probing with ^13^C-labelled diatom polysaccharide-rich microgels conducted previously at the site showed that *Cladosporium* assimilate phytoplankton-produced organic matter [32]. *Cladosporium* isolated from the site use the phytoplankton polysaccharide laminarin as a growth substrate [32], which is a major component of marine particulate organic carbon [33]. Marine fungal-phytoplankton organic matter interactions such as this are widespread [34], with taxa likely increasing in abundance when phytoplankton-derived substrates are highest. Studies at the Helgoland Roads long-term ecological research station in the German Bight (North Sea) have also indicated that fungal diversity and abundance may be associated with phytoplankton abundance in coastal marine waters [9,35]. Over a 1-year survey, Banos *et al*. [9] showed that the number of fungal operational taxonomic units was minimal during periods when phytoplankton abundance was high. Through a novel Catalyzed Reporter Deposition Fluorescence *in situ* Hybridization (CARD-FISH) protocol for absolute fungal quantification, Priest *et al*. [35] showed that fungal diversity is richest when abundance is minimal prior to the spring phytoplankton bloom when fungal abundance increases. Positive relationships between phytoplankton biomass, fungal abundance and activity also occur in the open ocean [36].

Interpretation of temporal dynamics in other plankton groups is supported by established empirical biological and ecological understanding (e.g. diatoms use silicon and other macronutrients to form major blooms in spring when conditions are favourable [31] and many *Flavobacteriales* bacterioplankton by degrading diatom produced polysaccharides increase in abundance during spring blooms [37]). Conversely, the fundamental biology and ecology of marine fungi remain relatively poorly understood compared to other marine microbial groups [38], and therefore, the exact mechanisms governing abundance patterns are yet to be established. The recurrence patterns shown here represent an important step towards building an ecosystem-level view of fungi in coastal marine ecosystems. Coastal marine fungi such as *Cladosporium* and *Metschnikowia* likely increase in abundance in response to seasonal fluctuations in diversity and abundance of other plankton (i.e. phytoplankton as a source of organic matter, and zooplankton as hosts) and cause the observed changes in apparent fungal diversity. As with other plankton groups, biotic interactions are presumably major factors in determining marine fungal diversity [9], which may account for the significant yet low impact of physico-chemical parameters (e.g. SST, nitrate/nitrite) on diversity we report here. Without a better biological and ecological understanding of marine fungi, interpretation of correlation analysis of metabarcoding diversity data is limited and more work is needed, especially on the major recurring taxa we show here.

In addition to patterns in major marine taxa, we also observed seasonality within low relative abundance taxa, many of which have a non-marine origin. Autumn, winter and spring-associated taxa include leaf litter (e.g. *Clitocybe*) and dead wood (e.g. *Trametes, Armillaria*) saprotrophs, as well as the freshwater hyphomycete *Tetracladium*. The most probable source of these taxa is deciduous woodland alongside rivers feeding into area, but the cause of the seasonal signal is uncertain and potentially the result of (i) increased abundance in the source environment; (ii) increased export of fungi-rich ‘transport’ substrates such as leaf litter or (iii) seasonally released spores. The first of these is likely, with our observations broadly corresponding to known patterns of activity in forest floor fungi. In temperate forests, activity of saprotrophic fungi in leaf litter is highest during the autumn and winter [39–41] with leaf litter accumulated in autumn depleted of readily labile organic compounds by early summer [41]. Aquatic Hyphomycetes are also typically associated with decaying deciduous leaf litter [42] with seasonality linked to substrate availability [43]. Increased rate of substrate export is also supported. Leaf litter is a primary source of allochthonous organic matter to rivers and streams [44] and increased riverine flow during autumn, winter and spring could lead to seasonally pulsed increases in fungi-rich material. Seasonal transport of larger lignin-rich particles could also account for the seasonal occurrence of deadwood decomposers which can display multi-annual as opposed to seasonal patterns of succession [45]. Evidence for seasonal sporulation is harder to establish, with different patterns occurring for different taxa. For example, more species of wood decomposing fungi fruit throughout the year while leaf litter fungi fruit in autumn [46]. The observed seasonality in the *Lichina* eDNA signal at Station L4 is not easily explained because, as with other marine fungi, their biology and ecology is relatively poorly understood compared to non-marine lichens. Our results here are possibly related to as yet uncharacterized phenology in *Lichina* reproduction, potentially with the controlled release of spores from enclosed ascocarps which are a characteristic trait of lichens that are regularly submerged in seawater [47]. It is likely that all the above mechanisms interact to generate the observed patterns, and further exploration of how seasonal patterns of abundance and activity of non-marine taxa in their native environment relate to corresponding eDNA signals in the coastal marine environment is necessary.

Metabarcoding of eDNA samples is currently the main culture-independent approach to assess fungal diversity and the ITS region the most widely used marker [48]. The choice of ITS region marker is especially important for this study given our aim of assessing diversity at the community and genus level. Despite the patterns we report here, there are caveats associated with eDNA metabarcoding-based approaches. With all eDNA-based assessments of diversity, we cannot be sure what type of material that eDNA is extracted from because it is possible that some eDNA signals may be from inactive material, such as spores (as discussed above). As with all PCR-based approaches, primer biases could be impacting amplification, and the copy number of the amplified region could vary between taxa and therefore impact the interpretation of relative abundance. Fortunately, as all samples are treated equally, any potential biases will be present across all samples so that the observed long-term intra- and inter-annual patterns we show are likely valid.

Of the 351 genera identified in this study from the eDNA-based survey of the coastal marine site, very few obligate marine taxa were identified (electronic supplementary material, figure S2), such as representatives of the iconic order Lulworthiales [49] and family Halosphaeriaceae [50]. The reasons for this are unclear; both Lulworthiales and Halosphaeriaceae belong in the Sordariomycetes which are otherwise frequent in the dataset. Their poor representation here could in part be due to current lack of suitable ITS reference sequences for obligate marine fungi in databases. It is also possible that these often seaweed and seagrass-associated fungi are not detectable in planktonic marine ecosystems; however, this seems unlikely given that we have detected eDNA from shore line (e.g. *Lichina*) and terrestrial woodland fungi. We focused on Ascomycota and Basidiomycota because they are the two major marine fungal groups generally in the oceans [7] and specifically at Station L4 [8]; however, we acknowledge that other fungal groups such as the Chytridiomycota are present in marine ecosystems, including at Station L4 [8], and probably have important roles such as parasitism at specific times.

In conclusion, seasonality is an established feature of marine plankton ecosystems in general, and in the light of our results, we propose that fungi should also be incorporated into the canonical view of the plankton calendar. Our approach has identified consistent, and in some cases long-term, potential key players in the structure and function of coastal marine ecosystems, including known saprotrophs involved in processing phytoplankton-derived organic matter (i.e. *Cladosporium*) and zooplankton parasites (i.e. *Metschnikowia*). Even though we show clear patterns in marine fungal diversity that are analogous with other microbial plankton groups, we shine a light on many as-yet functionally unknown players in the context of coastal marine ecosystems.

## Data Availability

Raw sequencing reads are available from NCBI Sequence Read Archive (SRA) under BioProject PRJNA865278. Additional data files are available in the electronic supplementary material [51].
